# Transgender men's preferences when choosing obstetricians and gynecologists

**DOI:** 10.1186/s13584-022-00522-z

**Published:** 2022-02-11

**Authors:** Dror Lifshitz, Iris Yaish, Gal Wagner-Kolasko, Yona Greenman, Yael Sofer, Sharon Alpern, Asnat Groutz, Foad Azem, Hadar Amir

**Affiliations:** 1 Department of Obstetrics and Gynecology, Sheba Medical Center, Tel Hashomer, Affiliated to the Sackler Faculty of Medicine, Tel Aviv University, Tel Aviv, Israel; 2 Institute of Endocrinology, Metabolism and Hypertension, Tel Aviv Sourasky Medical Center, Affiliated to the Sackler Faculty of Medicine, Tel Aviv University, Tel Aviv, Israel; 3 Department of Family Medicine, Clalit Gan-Meir LGBT Clinic, Tel Aviv District, Israel; 4 Sara Racine IVF Unit, Department of Obstetrics and Gynecology, Lis Maternity Hospital, Tel Aviv Sourasky Medical Center, Affiliated to the Sackler Faculty of Medicine, Tel Aviv University, Tel Aviv, Israel; 5 Department of Obstetrics and Gynecology, Chaim Sheba Medical Center (Tel Hashomer), Ramat Gan, Israel

**Keywords:** Transgender men, Obstetricians/gynecologists, Gender, Tolerant, Sexual and gender minorities

## Abstract

**Background:**

Transgender men are a marginalized population with unique health care needs. However, their usage of health services is low because of considerable discrimination. A major factor in their avoidance is patient-provider interactions.

**Methods:**

This cross-sectional study included 102 transgender men who anonymously completed a 55-item questionnaire in clinic, between 10/2017 and 01/2019. In addition, 92 transgender women filled out the part about family physician’s preferences. We examined which characteristics transgender men prefer in their obstetricians/gynecologists in order to promote their usage of healthcare services.

**Results:**

A small majority of the transgender men (54.1%) had no gender preference for their obstetrician/gynecologist, while 42.9% preferred a female obstetrician/gynecologist and 3.1% preferred a male obstetrician/gynecologist. Most transgender men with a same-gender preference preferred female obstetricians/gynecologists for both invasive procedures (e.g., pelvic examination, 97.4%) and non-invasive procedures (e.g., cesarean section, 60%). The reasons for preferences regarding invasive procedures were feeling comfortable, embarrassment and feeling that female obstetricians/gynecologists are gentler. Transgender men who preferred female obstetricians/gynecologists ranked ability (90.5%), sexual tolerance (92.9%) and gender identity tolerance (90.5%) as the top three desirable qualities of obstetricians/gynecologists, while the responders who did not prefer female ranked ability (94.6%), experience (92.9%) and knowledge (92.9%) as the top three qualities. Transgender men with female preferences considered female obstetricians/gynecologists to be more accepting of gender identity compared to the responders that did not prefer females (47.5% vs. 9.1%, *P* < .001)..

**Conclusion:**

A small majority of the transgender men exhibited no gender preference when choosing an obstetrician/gynecologist, although 42.9% preferred females. The latter choice was associated with the assumption that female obstetricians/gynecologists are more tolerant towards their transgender men patients. Educating the medical staff about their special needs and establishing dedicated SGM centers staffed with high percentages of female healthcare providers are highly recommended.

## Background

Transgender individuals are defined as those with a discrepancy between their sex assigned at birth and their personal gender identity (cis-centric) [1], a situation which often leads to severe mental distress (gender dysphoria). They are estimated to comprise around 0.5–0.6%of the population in the U.S. [2] with youth population approximately 2% [3], and reportedly range as high as 1.2% in other countries worldwide [4]. Transgender people have unique health issues compared to individuals whose gender identity matches the sex they were assigned at birth (cisgender) [1, 4], including a higher prevalence of risky health behaviors (smoking, alcohol and drug use) [5], mental health problems (depression, anxiety, and suicidality), and HIV and other sexually transmitted infections [6]. The poor health outcomes in this marginalized population have been explained by structural (e.g., laws and policies), interpersonal (e.g., provider discrimination) and individual (e.g., provider education and knowledge) barriers to healthcare [7]. Public awareness about transgender people has recently grown considerably, and the field of transgender health is experiencing a corresponding surge in interest on the part of policymakers and health providers. The World Health Organization has identified transgender people as comprising a population with high vulnerability and specific health needs that need to be addressed [8]. In 2016, the Obama administration expanded Sect. 1577, which prohibits discrimination by any federal health program on activity on the grounds of race, color, national origin, sex, age, or disability, to include prohibition of discrimination based on gender [9].

Providing transgender individuals adequate medical care is even more complex when it comes to procedures that are perceived by transgender patients as being invasive and cause them discomfort and distress [10]. Transgender men (individuals who were labelled as being females at birth but have a male gender identity) whose reproductive organs have not been removed require gynecological surveillance, including screening examinations, in order to prevent life-threatening medical conditions. Additionally, transgender men who choose to conceive, be pregnant and give birth are subject to the routine gynecological examinations. The American College of Obstetricians and Gynecologists called on obstetricians/gynecologists to help eliminate barriers for transgender men by creating nondiscriminatory practices, assisting with gender transition, and providing transgender-appropriate and comprehensive healthcare [11]. However, the obstetricians/gynecologic needs of transgender men are not taken care of and they use less the obstetric/gynecologic services, such as fertility preservation [10–12].

A key approach in today's healthcare is patient-centered care which acknowledges the patient’s needs and preferences. This is especially important for populations with unique health needs and for areas of medicine that require a pelvic examination. The factors that affect the patient’s choice of an obstetrician/gynecologist have recently been under investigation. Several papers that examined the influence of the physician’s gender in those choices among non-sexual and gender minorities (SGM) reported conflicting findings: some reported a clear preference for female physicians in the cisgender population [13], while others stated that the gender of their physician was not an important consideration when choosing an obstetrician/gynecologist [14]. Moreover, the choice of a female obstetrician/gynecologist was consistently more common among religious and ethnic minorities, primarily due to a sense of embarrassment during a pelvic examination [15]. Similarly, lesbians were found to prefer a female obstetrician/gynecologist because they felt more comfortable and described them as being gentler, kind, understanding, open and tolerant compared to male obstetricians/gynecologists [16, 17].

It is incumbent among medical providers to enable and encourage transgender men to obtain the medical care that they require. The aim of the current study was to explore transgender men’ preferences, including gender, when choosing their obstetricians/gynecologists, and to identify the factors that need to be modified in the healthcare system in order to promote greater accessibility and use of appropriate medical care of these individuals.

## Methods

### Ethical approval

This study was approved by the institutional review board of the Tel Aviv Sourasky Medical Center (TASMC) (#0455-17-TLV).

### Study population and participant recruitment

In light of the anticipated difficulty in recruiting transgender people [18], we chose to perform the study in two clinics that provide services mainly to SGM patients. All the consecutive eligible patients aged 18 years and above were asked to fill out the anonymous questionnaire before entering a meeting with the physician. After collecting a total of 200 questionnaires, a similar number of questionnaires were analyzed in previous studies that we conducted among minority populations, the recruitment process was stopped.

Of 113 transgender men and 101 transgender women (individuals who were labelled male at birth but have a female gender identity) 102 transgender men (90% response rate) and 92 transgender women (91% response rate) were prospectively enrolled and included in our analyses. Four transgender men completed solely the first part of the questionnaire and therefore were included in this part of the survey only. The participants were referred to the Gender Clinic, Institute of Endocrinology, Metabolism and Hypertension of TASMC, a tertiary university-affiliated medical center, and to the Gender Clinic, Gan Meir Community Health Care Center, Clalit Health Services, Tel Aviv, Israel between October 2017 and January 2019.

The patients met with endocrinologists in the first clinic visit and with a family physician in the second visit. Patients who were referred to both clinics were asked to fill out the anonymous questionnaire before entering a meeting with the physician. Transgender men completed an anonymous questionnaire designed to assess gender preferences in choosing their healthcare providers, including obstetricians/gynecologists. Transgender women were asked to fill out the part about their family physician’s preferences. All patients received an explanation of the questionnaire from the nurse, filled out the questionnaire independently in the waiting room, and then put it through a slot in a closed box. The researchers emptied the box daily and transferred the data provided in the forms to a computerized database.

### Questionnaire for the transgender men

The researchers designed the study questionnaire of 55 items based on previous studies [13, 14]. The first part of the questionnaire included basic sociodemographic and clinical information, and those items were answered by circling the selected choice. The second part included questions about gender preferences of provider, gender preferences for different medical situations, such as pelvic examinations, pregnancy follow-up visits, gynecologic surgeries, in vitro fertilization treatment or any other consultation for obstetric or gynecologic issues. The transgender men were further asked to identify specific characteristics of the obstetrician/gynecologist related to their gender preference by circling the word “male”, “female” or “none”. Lastly, each participant was also asked to circle characteristics he considered to be the most important in choosing his obstetrician/gynecologist from a list of 16.

### Statistical analysis

Descriptive statistics were computed as mean and standard deviation (SD) for continuous variables and as frequencies for categorical variables. Significance was tested with the t-test, Mann–Whitney U test, χ2 and Fisher’s exact test as needed. The data are summarized as mean ± SD, or number of responders (percentage) according to the variables. *P* < 0.05 was considered statistically significant. All statistical analyses were performed with IBM SPSS Statistics version 25.

## Results

The study population was comprised of 102 transgender men (mean age 27 ± 8.16, range 18–62 years). In addition, 92 transgender women (mean age 28 ± 8.2, range 18–57 years) filled out the part about family physician’s preferences. No significant socio-demographic or clinical differences were found between the two groups except for upper body surgery (Table 1). A significantly higher rate of upper body surgery was performed among transgender men compared to transgender women (48% vs. 20.7%, respectively, *P* < 0.001).

**Table 1:** Demographic and clinical characteristics of the 194 transgender responders to the study survey

Characteristic	Transgender women (n = 92) n, (%)	Transgender men (n = 102) n, (%)	*P* value
Age, mean (SD) (range)	28 (8.2) (18–57)	27 (8.16) (18–62)	NS
Origin			
Israel	69 (82.1)	85 (84.2)	
Other	15 (17.9)	16 (15.8)	NS
Religion			
Jewish	76 (82.6)	89 (87.3)	
Other	16 (17.4)	13 (12.7)	NS
Religious status			
Secular	72 (79.1)	81 (82.7)	
Religious	19 (20.9)	17 (17.3)	NS
Marital status			
Married or with a partner	19 (20.7)	35 (35.4)	
Single or divorced	73 (79.3)	64 (64.6)	NS
Children			
Yes	7 (7.6)	8 (8.1)	
No	85 (92.4)	91 (91.9)	NS
Education			
Primary school	10 (11.5)	6 (5.9)	
High school	50 (57.5)	61 (59.8)	
College degree or higher	27 (31)	35 (34.3)	NS
Employment			
Yes	61 (66.3)	64 (64.6)	
No	31 (33.7)	35 (35.4)	NS
Sexual orientation			
Heterosexual	54 (60.7)	43 (43.9)	
Homosexual	20 (22.5)	15 (15.3)	
Bisexual	12 (13.5)	33 (33.7)	
Asexual	3 (3.4)	7 (7.1)	NS
Psychiatric medication			
Yes	32 (35.6)	28 (28)	
No	58 (64.4)	72 (72)	NS
Gender-affirming hormone therapy			
None	15 (17.6)	22 (22.9)	
Less than one year	26 (30.6)	25 (26)	
1–5 years	39 (45.9)	39 (40.6)	
More than 5 years	5 (5.9)	10 (10.4)	NS
Upper body surgery			
Yes	19 (20.7)	49 (48)	
No	73 (79.3)	53 (52)	** < 0.001**
Lower body surgery			
Yes	7 (7.8)	4 (4)	
No	83 (92.2)	96 (96)	NS

Bold represents *P*-value under 0.05 was considered to be significant

As shown in Table 2, about one-half of each group of transgender women and men were routinely managed by male family physicians (47.3% and 42.4%, respectively, *P* = 0.194) and the other half by female family physicians (39.6% and 50.5%, respectively, *P* = 0.194). Most of the study participants had no gender preference for their family physician (59.6% of transgender women and 64.6% of transgender men, *P* = 0.242).

**Table 2:** Gender preference for family physician of 194 transgender responders to the study survey

Characteristic	Transgender women (n = 92) n, (%)	Transgender men (n = 102) n, (%)	*P* Value
Family physician’s gender (last 3 years)			
Male	43 (47.3)	42 (42.4)	
Female	36 (39.6)	50 (50.5)	
None	12 (13.2)	7 (7.1)	NS
Preferred gender for family physician			
Male	2 (2.2)	6 (6.1)	
Female	34 (38.2)	29 (29.3)	
None	53 (59.6)	64 (64.6)	NS

**Fig. 1:**
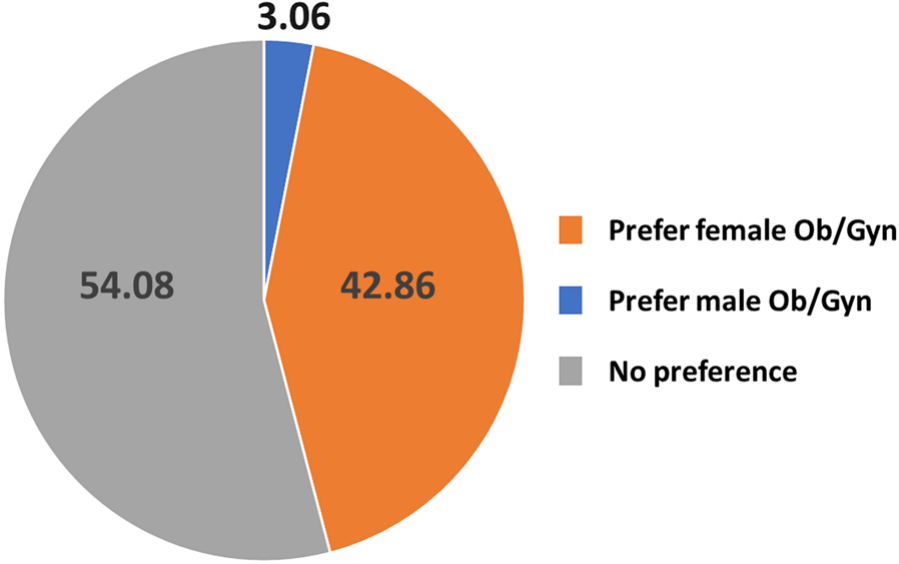
Gender preference for a male or female obstetrician/gynecologist (Ob/Gyn) among transgender men

We next explored the physician preferences of transgender men when choosing an obstetrician/gynecologist. Fifty-three (54.1%) had no gender preference and 42 (42.9%) preferred a female obstetrician/gynecologist, a higher figure than the 29.3% of them who preferred a female family physician. Only three (3.1%) replied that they preferred a male obstetrician/gynecologist (Fig. 1). The preference for a female obstetrician/gynecologist was more common among less-educated patients compared to a male obstetrician/gynecologist or no gender preference (Table 3). Primary school- and high school-educated students indicated a female preference (11.9% vs. 1.8% and 64.3% vs. 55.4%, respectively, *P* = 0.04), while college/university graduates did not (23.8% vs. 42.9%, *P* = 0.04). Preferring a female obstetrician/gynecologist was also more common among the same transgender men who preferred a female family physician compared to a male or no gender preference of family physician (38.3% versus 7.3%, respectively; *P* < 0.001).

**Table 3:** Demographic and clinical characteristics of 98 transgender men classified by their preference for a male or female obstetrician/gynecologist (Ob/Gyn)

Characteristic	Prefer female Ob/Gyn (n = 42)	Prefer male Ob/Gyn or no gender preference for Ob/Gyn (n = 56)	*P* Value
Age, mean (SD) (range)	26.7 (7.42) (18–50)	27.3 (8.52) (18–62)	NS
Origin			
Israel	37 (88.1)	45 (80.4)	
Other	5 (11.9)	11 (19.6)	NS
Religious status			
Secular	33 (80.5)	9 (16.1)	
Religious	8 (19.5)	47 (83.9)	NS
Marital status			
Married or with a partner	10 (25)	23 (41.8)	
Single or divorced	30 (75)	32 (58.2)	NS
Children			
Yes	5 (12.5)	3 (5.5)	
No	35 (87.5)	52 (94.5)	NS
Education			
Primary school	5 (11.9)	1 (1.8)	
High school	27 (64.3)	31 (55.4)	
College degree or higher	10 (23.8)	24 (42.9)	**0.04**
Employment			
Yes	26 (65)	36 (64.5)	
No	14 (35)	19 (34.5)	NS
Sexual orientation			
Mainly attracted to women	16 (40)	26 (48.1)	
Mainly attracted to men	4 (10)	9 (16.7)	
Bisexual	15 (37.5)	17 (31.5)	
Asexual	5 (12.5)	2 (3.7)	NS
Psychiatric medications			
Yes	14 (33.3)	14 (25)	NS
No	28 (66.7)	42 (75)	
Gender-affirming hormone therapy			
None	8 (20.5)	13 (23.6)	
Less than one year	15 (38.5)	9 (16.4)	
1–5 years	12 (30.8)	27 (49.1)	
More than 5 years	4 (10.3)	6 (10.9)	NS
Upper body surgery			
Yes	17 (40.5)	29 (51.8)	
No	25 (59.5)	27 (48.2)	NS
Lower body surgery			
Yes	0 (0)	4 (7.1)	
No	41 (100)	52 (92.9)	NS
Preferred gender for family physician			
Male	2 (3.3)	4 (7.3)	
Female	23 (38.3)	4 (7.3)	
None	35 (58.3)	47 (85.7)	** < 0.001**

Bold represents *P*-value under 0.05 was considered to be significant

*The values in the parentheses are percentages unless indicated otherwise

**Fig. 2:**
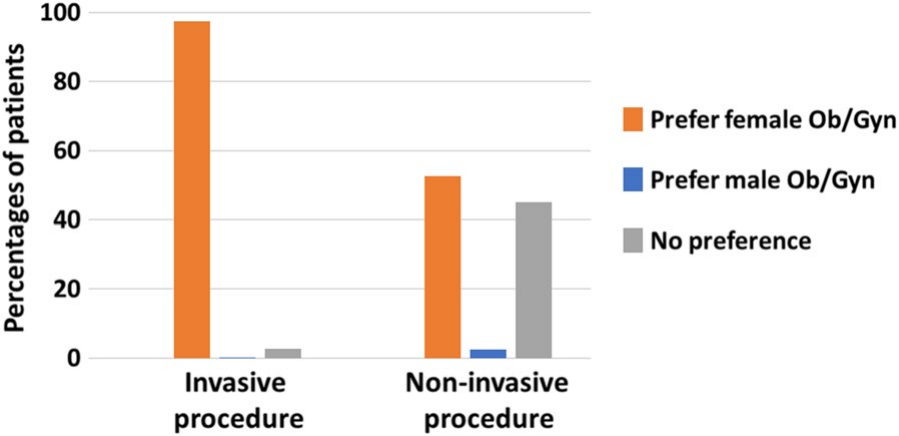
Preference for a male or female Ob/Gyn for invasive (pelvic examination) versus non-invasive (no pelvic examination) procedures among transgender men

Table 4 and Fig. 2 displays gender preferences for invasive versus non-invasive aspects of gynecological care. Transgender men who preferred a female obstetrician/gynecologist chose a female obstetrician/gynecologist for both invasive and non-invasive procedures compared to transgender men who did not (*P* < 0.001). Almost all of the transgender men who preferred female obstetricians/gynecologists preferred them for pelvic examinations (97.4%) and most (82.5%) preferred them for pregnancy follow-up procedures that are considered invasive. However, the preference for a female obstetrician/gynecologist was less pronounced when it came to non- invasive procedures, such as a caesarian Sect. (60%). Interestingly, the percentage of transgender men who preferred female obstetricians/gynecologists for surgical procedures such as gynecological surgery (52.5%) was lower than for non-surgical procedures, such as pregnancy follow-up (82.5%).

**Table 4:** Preference for a male or female obstetrician/gynecologist (Ob/Gyn) for invasive versus non-invasive Ob/Gyn procedures among transgender men

Characteristic	Prefer female Ob/Gyn (n = 42)	Prefer male Ob/Gyn or no Ob/Gyn gender preference (n = 56)	*P* Value
*Invasive procedure*			
Gender preference for pelvic examination			
Male	0 (0)	4 (7.5)	
Female	37 (97.4)	7 (13.2)	
None	1 (2.6)	42 (79.2)	** < 0.001**
Gender preference for gyn consultation			
Male	0 (0)	2 (3.7)	
Female	31 (77.5)	3 (5.6)	
None	9 (22.5)	49 (90.7)	** < 0.001**
Gender preference for pregnancy follow-up			
Male	0 (0)	1 (1.9)	
Female	33 (82.5)	3 (5.8)	
None	7 (17.5)	48 (92.3)	** < 0.001**
Gender preference for fertility preservation treatments			
Male	0 (0)	2 (3.7)	
Female	24 (60)	1 (1.9)	
None	16 (40)	51 (94.4)	** < 0.001**
*Non-invasive procedure*			
Gender preference for caesarean section			
Male	0 (0)	0 (0)	
Female	24 (60)	1 (1.9)	
None	16 (40)	51 (98.1)	** < 0.001**
Gender preference for gyn surgery			
Male	1 (2.5)	1 (1.9)	
Female	21 (52.5)	4 (7.4)	
None	18 (45)	49 (90.7)	** < 0.001**
Gender preference if no pelvic examination needed			
Male	2 (5.1)	2 (3.6)	
Female	22 (56.4)	2 (3.6)	
None	15 (38.5)	51 (92.7)	** < 0.001**

Bold represents *P*-value under 0.05 was considered to be significant

*The values in the parentheses are percentages unless indicated otherwise

The reasons for preferring a female obstetrician/gynecologist were then queried (Table 5). The major reason given by the transgender men who preferred a female over a male obstetrician/gynecologist was their feelings during a pelvic examination. They described feeling more comfortable with female obstetrician/gynecologist (100% vs. 13%, respectively, *P* < 0.001) and more embarrassed with male obstetrician/gynecologist (36.8% vs. 0%, *P* < 0.001), and judged the pelvic examination as being more gentle (69.2% vs. 5.4%, *P* < 0.001). They also considered female obstetricians/gynecologists as being more sympathetic than their male counterparts (57.9% vs. 9.3%, *P* < 0.001).

**Table 5:** Reasons for preference for a male or female obstetrician/gynecologist (Ob/Gyn) for specific Ob/Gyn procedures among transgender men

Characteristic	Prefer female Ob/Gyn (n = 42)	Prefer male Ob/Gyn or no Ob/Gyn gender preference (n = 56)	*P* Value
*Pelvic examination*			
More embarrassment			
Male	14 (36.8)	0 (0)	
Female	0 (0)	2 (3.7)	
No preference	24 (63.2)	52 (96.3)	** < 0.001**
More comfortable			
Male	0 (0)	5 (9.3)	
Female	38 (100)	7 (13)	
No preference	0 (0)	42 (77.8)	** < 0.001**
More gentle			
Male	0 (0)	6 (10.7)	
Female	27 (69.2)	3 (5.4)	
No preference	12 (30.8)	47 (83.9)	** < 0.001**
*Physician’s characteristics*			
More sympathetic			
Male	0 (0)	5 (9.3)	
Female	22 (57.9)	5 (9.3)	
No preference	16 (42.1)	44 (81.5)	** < 0.001**
More patient			
Male	0 (0)	3 (5.4)	
Female	20 (50)	3 (5.4)	
No preference	20 (50)	50 (89.3)	** < 0.001**
Spends more time with patient			
Male	0 (0)	3 (5.5)	
Female	5 (12.8)	0 (0)	
No preference	34 (87.2)	52 (94.5)	**0.01**
*Physician’s professionalism*			
More understanding of transgender health			
Male	0 (0)	2 (3.6)	
Female	15 (38.5)	3 (5.4)	
No preference	24 (61.5)	51 (91.1)	** < 0.001**
More knowledgeable in transgender health			
Male	0 (0)	1 (1.8)	
Female	12 (30.8)	2 (3.6)	
No preference	27 (69.2)	53 (94.6)	** < 0.001**
Better physician in general			
Male	0 (0)	1 (1.8)	
Female	11 (28.2)	2 (3.6)	
No preference	28 (71.8)	53 (94.6)	** < 0.001**
More accepting of sexual preference			
Male	0 (0)	1 (1.8)	
Female	16 (40)	6 (10.7)	
No preference	24 (60)	49 (87.5)	** < 0.001**
More accepting of gender identity			
Male	1 (2.5)	1 (1.8)	
Female	19 (47.5)	5 (9.1)	
No preference	20 (50)	49 (89.1)	** < 0.001**

Bold represents *P*-value under 0.05 was considered to be significant

*The values in the parentheses are percentages unless indicated otherwise

**Fig. 3:**
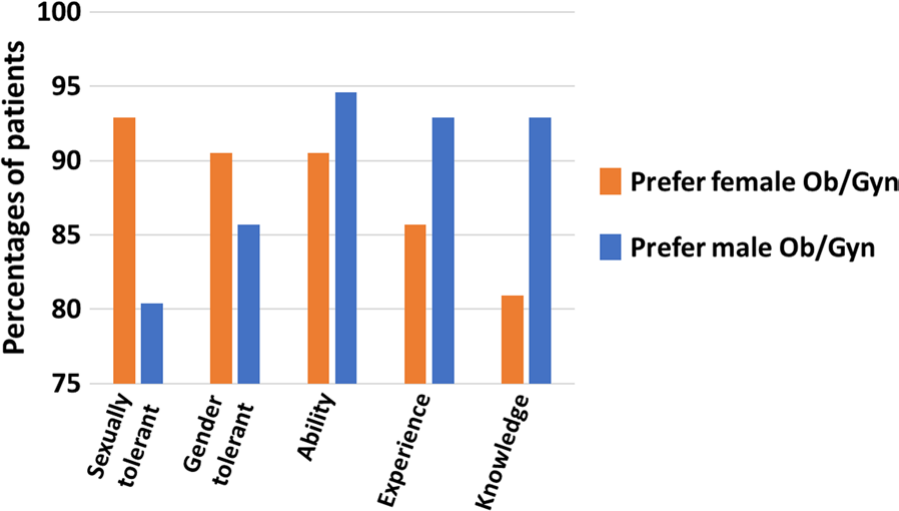
Comparison between transgender men who preferred female Ob/Gyn versus male Ob/Gyn for the top 3 factors affecting their choice

Among the top three factors that influenced transgender men regarding their preference in choosing an obstetrician/gynecologist, only one, “ability”, was chosen by the vast majority of both groups (90.5% and 94.6%) (Table 6; Fig. 3). The other two parameters differed according to the subject’s gender preference of physician: transgender men who preferred female obstetricians/gynecologists highly ranked “sexually tolerant” (92.9%) and “gender tolerant” (90.5%), while transgender men who did not prefer female obstetricians/gynecologists ranked “experience” and “knowledge” (92.9% for both) as the other two most important characteristics. The gender of the obstetrician/gynecologist was the only parameter that was significantly different between the two groups (33.3% vs. 3.6%, respectively, *P* < 0.001).

**Table 6:** Characteristics and percentages of the 16 factors ranked by transgender men as affecting their choice for a female or male obstetrician/gynecologist (Ob/Gyn)

Characteristic	Prefer female Ob/Gyn (n = 42)	Prefer male Ob/Gyn or no Ob/Gyn gender preference (n = 56)	*P* Value
Demographics			
Age	4 (9.5)	3 (5.4)	NS
Sex	4 (33.3)	2 (3.6)	** < 0.001**
Religion	5 (11.9)	1 (1.8)	NS
Marital status	0	0	
Parental status	0	1 (1.8)	NS
Professional skills			
Ability (professional)	38 (90.5)	53 (94.6)	NS
Experience	36 (85.7)	52 (92.9)	NS
Knowledge	34 (80.9)	52 (92.9)	NS
Reputation	15 (35.7)	18 (32.1)	NS
Qualifications			
Board certification	15 (35.7)	27 (48.2)	NS
Hospital affiliation	2 (4.8)	4 (7.1)	NS
University affiliation	1 (2.4)	2 (3.6)	NS
Other qualities			
Personality	31 (73.8)	40 (71.4)	NS
Availability	19 (45.2)	27 (48.2)	NS
Sexually tolerant	39 (92.9)	45 (80.4)	NS
Gender tolerant	38 (90.5)	48 (85.7)	NS

Bold represents *P*-value under 0.05 was considered to be significant

*The values in the parentheses are percentages unless indicated otherwise

## Discussion

Many studies on SGM and health system issues have been published, but they did not identify the parameters most important to transgender men in their choice between female and male obstetricians/gynecologists. This study was designed to fill that gap. Transgender patients are a gender minority that confronts serious discrimination when approaching health services. In 2011, The National Discrimination Survey revealed that a high percentage of transgender responders experienced discrimination in various sectors of health services, such as a 24% discrimination rate at the physician’s office [19]. Recent studies confirmed the negative attitude of the health system towards them that lead to their healthcare needs being unmet [9–11, 19]. The biggest obstacle involved in transgender avoidance of healthcare systems was reported as being patient-provider interactions [20]. Healthcare providers have been implicated in low utility of healthcare services by other minorities as well [21]. Many studies examined which feature minorities prefer in their physician and identified same-gender preference as being a major one [15, 16]. In addition to routine care, the healthcare needs of transgender people include unique treatments, such as gender-affirming hormone therapy, body-altering surgeries, psychological support, and others [22]. Family physician are the first line healthcare providers that encounter the transgender patient, manage their healthcare needs and connect them with other specialists as required [22]. There are enormous barriers when transgender people confront discriminative behavior and lack of knowledge on the part of the primary physician [19, 20], and those barriers interfere with the reception of appropriate quality care [23]. Similar to studies conducted among diverse minorities [15, 16, 24], we did not find a major bias against male or female family physicians among both transgender men and women. Same-gender preference for family physician is not crucial among SGM, presumably because other parameters are more relevant to them [16, 24, 25].

The obstetrician/gynecologist has a significant role in transgender men’s health. The needs of the transgender individuals are unique, such as the management of the gender-affirming hormone therapy, fertility consultation, gynecologic follow-up, etc. [19, 26, 27]. Many transgender people reportedly experience discrimination on the part of their obstetricians/gynecologists, and many find that experience a trigger of dysphoria [19, 26–28]. The outcome is their avoidance of treatment despite their special needs and risk factors [19, 26–28]. The discrepancy between the high needs and low usage of obstetric/gynecologic services among minorities has previously been linked with the gender of their provider [15–17]. For example, Israeli Druze women responders described bias based on their traditional and religious beliefs [15]. Contemporary pro-active women’s groups designated same-gender preference as part of their feminist dogma [13], and sexual minorities due to greater tolerance of the same-gender providers to their sexual orientation [16, 17].

Our working hypothesis that the invasive nature of the obstetric/gynecologic procedures would motivate transgender men to prefer a female obstetrician/gynecologist did not materialize and a small majority of the responders had no preference for obstetrician/gynecologist. Still, it should be noted that a much higher percentage of the transgender men (42.9%) did prefer a female obstetrician/gynecologist compared with those who preferred a male (3.1%). Moreover, excluding the responders with no preference and exploring only the responders that have preference results in 93.3% of transgender men that prefer a female obstetrician/gynecologist while only 6.7% preferred a male obstetrician/gynecologist. It should be noted that our study population is not homogeneous since it is composed of responders in different stages of their transition process. Some transgender men might be similar to cisgender men who preferred a male physician for urological care [29]. In agreement with our results, Ettner et al. did not find clear preference for same-gender surgeons by transgender people undergoing gender-affirming surgery despite the invasive nature of that surgery [30].

One of the main reasons for same-gender preference of other minorities was based on how they felt during pelvic examinations, e.g., more comfortable and less embarrassed [15, 16]. As a result, their preference for a female obstetrician/gynecologist was restricted to invasive procedures [15, 16]. The reason our responders gave for their same-gender preference during invasive procedures was similar to that other minorities, including feeling more comfortable, less embarrassed and feeling that a female obstetrician/gynecologist is gentler. However, their bias was not limited to invasive procedures but applied to non-invasive ones as well. Previous studies have demonstrated that transgender people’s visit to an obstetrician/gynecologist is associated with anxiety resulting from factors other than feeling during invasive procedures. In a survey of transgender men, 92% reported anxiety regarding receiving gynecological care due to the following reasons: encountering gendered forms (50%), sitting in a waiting room with cisgender women (54%), being misgendered (59%), having to educate the provider about transgender issues (70%) and undergoing the gynecologic exam itself (86%) [28]. Dutton et al. found that all of their transgender men study participants did not like receiving gynecological care not only because of the exposure of personal body part, but also because the visit itself was accompanied by an extreme emotional conflict between self-perceptions and physical anatomy [26]. The reason of our responders for their same-gender preference during non-invasive procedures presumably result from the emotional issues that emerge during the visit at the obstetrician/gynecologist.

Our transgender men responders that prefer female significantly preferred gender tolerant and sexually tolerant providers above all other factors with the exception of professional ability. Unlike our responders, religious and ethnic minorities added professional skills as interpreted by the parameters of experience and knowledge to that of ability [15], although the priorities of other SGM were influenced by their sexual orientation and gender identity [16, 17, 25, 31]. Bisexual, queer or lesbian women were reported to prefer providers who were open-minded and friendly toward people of different sexual orientations [32]. SGM youth responders ranked provider qualities and interpersonal skills, such as being respectful, non-judgmental and treating SGM equally, higher than provider knowledge and experience [24]. Frecker et al. found that the transgender men responders avoided undergoing gynecological treatment due to transgender-non-friendly and non-transgender-knowledgeable healthcare providers [28]. Having a tolerant physician apparently pales other characteristics and emphasizes the importance of such qualities to transgender men that prefer female. The association between the healthcare provider's gender and level of tolerance has already been established. SGM responders demonstrated preference for female family physicians due to their kinder, more accepting and more open character [23]. Abdessamad et al. demonstrated higher Homosexuality Attitude Scale scores among women [33]. In our study, the transgender men that prefer female considered female obstetricians/gynecologists as being far more accepting of gender identity (47.5%) compared to male obstetricians/gynecologists (2.5%). Their preference was significantly higher than responders who did not prefer female obstetricians/gynecologists. Same-gender preference of our responders might be ascribed to their conception that female obstetricians/gynecologists are more tolerant than male obstetricians/gynecologists.

To the best of our knowledge, this is the first study that addresses transgender men preferences when choosing an obstetrician/gynecologist. However, it is not without its’ limitation: (1) No published data about the sociodemographic and clinical profiles of transgender persons in Israel are available, which consequently limits the possibility to examine whether our study group represents the transgender population inside and outside Israel. However, based on previous studies we can conclude that some of the characteristics including religious status [34], education [35], employment [36, 37], sexual orientation [34], mental disorders [35, 36], and gender-affirming surgeries [38, 39], are representative. Furthermore, most of the transgender adolescents and adults nationwide are referred to our hospital because it is part of a national center for transgender health medicine. This enables the adequate representation of all strata of the transgender population. In addition, the study was conducted in two clinics, in and out of the hospital, to increase the population's representation. Still, we are aware of this weakness thus calling for further studies on larger populations. (2) The study was conducted in only two centers, therefore, interpersonal relationships with physicians might influence patient preference. However, both clinics are large and enable exposure to a variety of physicians. (3) Both centers are located in the center of Tel Aviv, the most liberal area in Israel. Nonetheless, as our study is relatively large, and was performed, as already mentioned, in a multi-disciplinary clinic that serves as the national referral center for gender dysphoric patients from across Israel it very likely reflects the preferences of this unique population. (4) Most of the patients were exposed to female physicians in the study clinics (75% female physicians). It is possible that if the exposure rate to male physicians was higher, the results would have changed. (5) Respondents were not asked if they prefer transgender clinician. This knowledge is significant and might influence the results. Further studies that include this information are desirable.

We found that small majority of the transgender men do not exhibit preference for obstetricians/gynecologists' gender, though their tendency was to prefer female obstetrician/gynecologist. Transgender men tendency was associated with the preference for tolerant obstetrician/gynecologist and the assumption that female obstetricians/gynecologists are more accepting and tolerant for gender and sexual minority patients.

Their unique health requirements and the discriminatory environment they encounter in healthcare facilities drive transgender men to seek an open-minded and tolerant obstetrician/gynecologist that accepts their gender identity. Educating the medical staff about their special needs and establishing dedicated SGM centers staffed with high percentages of female healthcare providers are highly recommended.

### Policy implications

Despite the increased understanding of the value of treating transgender patients in a knowledgeable, mindful and supportive manner, transgender health policies and their healthcare delivery issues are still lacking. There is negligible public and governmental awareness of the crucial needs of the transgender population in general, and specifically of their unique healthcare needs that are different from traditional ones [9–11, 19, 26–28]. Transgender issues are either not included or have not been updated in medical studies [19, 40]. There is a dearth of education on the issues that concern transgender people’s health, and even more so with regard to issues of gynecology and reproduction [41, 42]. Medical providers, such as obstetricians/gynecologists, acquire their knowledge from several limited resources (World Professional Association for Transgender Health-Standards of Care and Diagnostic and Statistical Manual of Mental Disorders), and are usually not aware of the unique physical and mental needs of their transgender patients [28, 40, 43, 44]. Hospitals and healthcare centers lack policies and fundamental knowledge about the transgender population’s healthcare issues [9–11, 19, 26–28], often preferring to avoid them altogether.

The transgender healthcare field of care is still in its infancy, but there are considerable efforts to promote the rights of transgender people to receive equal and competent healthcare services. Formal recommendations for specific education objectives regarding transgender healthcare were published by the Council of Resident Education in Obstetrics and Gynecology in the USA [45]. The American College of Obstetricians and Gynecologists has produced recommendations on care, support, education and awareness for obstetrician and gynecologist specialists regarding their transgender patients [46]. Additionally, there are universities in the USA and Canada that include updated and expanded curriculum on the care of transgender populations in their undergraduate and graduate medicine programs [45, 47–50]. In the UK, University College London and Bristol Medical School have implemented sessions to raise awareness of SGM health inequalities [51]. In Israel, the Tel Aviv University includes transgender care in its obstetricians/gynecologists continuing medical education training [52]. There are updated sources of information about transgender care, including medical books, e.g., dedicated chapters are included in the new edition of Speroff's Clinical Gynecologic Endocrinology and Infertility [53]. New web-based resources include SGM educational materials and workshops online that provide updated knowledge and education about the transgender population’s healthcare [44, 54, 55]. Several new dedicated SGM interdisciplinary healthcare centers that are composed of transgender-friendly interdisciplinary healthcare providers have also been established [56, 57]. The Tel Aviv Medical Center (Tel Aviv, Israel) recently founded a national center for transgender health medicine which includes transgender-friendly endocrinologists, plastic surgeons, skin specialists, psychologists and obstetrician/gynecologist specialists [58].

We contend that certain policy changes are warranted in order to encourage greater accessibility by transgender people to the healthcare system. We urge increasing public awareness and establishing governmental policies to encourage additional changes in approaches to healthcare issues, such as campaigning to educate providers, establishing enlightened hospital policies, creating dedicated centers, etc. We suggest creating more dedicated SGM-friendly clinics and centers that will staff tolerant and trans-experienced experts, including obstetricians/gynecologists with high percentages of female healthcare providers. We strongly recommend establishing formal protocols and guidelines regarding transgender people’s healthcare that will be an integral part of the undergraduate and graduate medical students' curriculum, in addition to periodical mandatory training programs and workshops for physicians and experts in transgender medicine. The overall aim is to achieve basic knowledge among the entire population of healthcare providers that will enable them to provide basic mindful and tolerant treatment to transgender patients. Such interventions have already proven to be successful and shown to have improved providers' attitudes and professional skills in the USA and UK [55, 59]. We urge the promotion and financial support of these efforts through fellowships and the inclusion of sub-specialties in SGM health programs that will allow young physicians to become experts in SGM medicine. For example, obstetrician/gynecologist experts in fertility will be able to gain advanced knowledge and expertise in SGM medicine and apply these skills to provide suitable treatments to transgender individuals (e.g., fertility preservation or pregnancy follow-ups for transgender men) [60]. We believe that the combination of mandatory basic training to all general healthcare providers together with elective fellowship/sub-specialization training to healthcare experts will enable transgender patients to receive compassionate, competent, and appropriate healthcare.

## Conclusions

Transgender men comprise a marginalized minority that their usage of healthcare services is low despite their high levels of medical needs. A major factor in their avoidance to seek such services is patient-provider interactions. Most transgender men that reported gender preference when choosing an obstetrician/gynecologist preferred female physicians. Their choice was associated with the assumption that female obstetricians/gynecologists are more tolerant towards their transgender male patients. In order to overcome such avoidance and encourage greater accessibility to the healthcare system, there is a need to increase the awareness of the population, specifically, that of healthcare providers, to the unique physical and mental healthcare needs of transgender patients. Educating and training the medical staff about those unique needs are mandatory in order to overcome the paucity of education on these issues, and to turn around the discriminatory environment confronted by the transgender population. Establishing dedicated SGM interdisciplinary healthcare centers staffed with tolerant and trans-experienced experts, including obstetricians/gynecologists with high percentages of female healthcare providers, is highly recommended.

## Data Availability

Data are available upon request.

## Abbreviations

LGBTQ: Lesbian, gay, bisexual, transgender and queer
OB\GYN: Obstetrician/gynecologist
SD: Standard deviation
SGM: Sexual and gender minorities
